# Immune repertoire fingerprinting by principal component analysis reveals shared features in subject groups with common exposures

**DOI:** 10.1186/s12859-019-3281-8

**Published:** 2019-12-04

**Authors:** Alexander M. Sevy, Cinque Soto, Robin G. Bombardi, Jens Meiler, James E. Crowe

**Affiliations:** 1 Chemical & Physical Biology Program, Vanderbilt University, Nashville, TN 37235 USA; 2 Center for Structural Biology, Vanderbilt University, Nashville, TN 37235 USA; 3 Vanderbilt Vaccine Center, Vanderbilt University Medical Center, Nashville, TN 37232 USA; 4 Department of Pediatrics, Vanderbilt University Medical Center, Nashville, TN 37232 USA; 5 Department of Chemistry, Vanderbilt University, Nashville, TN 37235 USA; 6 Department of Pathology, Microbiology and Immunology, Vanderbilt University Medical Center, Nashville, TN 37232 USA

**Keywords:** Immune repertoire analysis, Principal component analysis, Antibody sequencing, Repertoire dissimilarity index

## Abstract

**Background:**

Advances in next-generation sequencing (NGS) of antibody repertoires have led to an explosion in B cell receptor sequence data from donors with many different disease states. These data have the potential to detect patterns of immune response across populations. However, to this point it has been difficult to interpret such patterns of immune response between disease states in the absence of functional data. There is a need for a robust method that can be used to distinguish general patterns of immune responses at the antibody repertoire level.

**Results:**

We developed a method for reducing the complexity of antibody repertoire datasets using principal component analysis (PCA) and refer to our method as “repertoire fingerprinting.” We reduce the high dimensional space of an antibody repertoire to just two principal components that explain the majority of variation in those repertoires. We show that repertoires from individuals with a common experience or disease state can be clustered by their repertoire fingerprints to identify common antibody responses.

**Conclusions:**

Our repertoire fingerprinting method for distinguishing immune repertoires has implications for characterizing an individual disease state. Methods to distinguish disease states based on pattern recognition in the adaptive immune response could be used to develop biomarkers with diagnostic or prognostic utility in patient care. Extending our analysis to larger cohorts of patients in the future should permit us to define more precisely those characteristics of the immune response that result from natural infection or autoimmunity.

## Background

Adaptive immune receptors on the surface of lymphocytes are the principal determinants of the adaptive immune response responsible for specific molecular recognition, necessary for a rapid and long-lived immune response to infection [1]. B cell encoded immunoglobulins are of particular interest due to their diversity and remarkable specificity. Immunoglobulin genes are formed by recombination events joining variable (V), diversity (D), and joining (J) genes to encode the variable region of an antibody sequence [2]. Recombination of different gene segments (V, D, and J gene segments for heavy chains, and V and J gene segments for light chains), along with addition of non-templated nucleotides at the junction between gene segments, heavy chain and light chain pairing, and somatic hypermutation, are all molecular processes responsible for generating immense diversity in the amino acid sequence of rearranged immunoglobulins. The total diversity of the antibody repertoire owing to these mechanisms has the theoretical potential to be 10^11–12^ in any given individual [2, 3] although recent studies have shown human antibody repertoires to be much smaller [4, 5]. Rapid advances in next-generation sequencing (NGS) have now made it possible to interrogate an individual’s repertoire directly through sequencing of antibody variable genes in B cells [6, 7].

Antibody repertoire sequencing has been used to analyze clonal lineages of antibodies in diverse settings, such as antibodies specific to HIV [8, 9] or influenza [10–12], as well as to characterize repertoires in patients with autoimmune disorders [13, 14]. However, in the absence of functional data about the specificity of individual clones, it is unclear how to best interpret antibody gene sequence data. In addition, it is difficult to compare repertoires between individuals to glean any meaningful data on how their antibody repertoires compare. Several groups have published methods to differentiate repertoires [15–17] and to predict characteristics of B and T cell repertoires based on features such as heavy chain complementarity-determining region 3 (CDRH3) length, amino acid composition, and germline gene usage [3, 18–20]. However, these methods use parameters derived from the primary data that have been computed from the high-dimensional data derived from antibody sequencing. We hypothesize that an unsupervised method that operates on the sequence data directly will improve accuracy and confidence when distinguishing between antibody repertoires. Previous methods have used principal components analysis (PCA) as an unsupervised approach to interpreting immune repertoire features [21–23].

In this work, we report a new method we refer to as “repertoire fingerprinting” that uses PCA of repertoire-wide V and J germline gene segment pairs to reduce each repertoire to a set of two components. The resulting PCAs can be analyzed to infer common and unique features between repertoires. We applied PCA to repertoire data for plasmablasts in blood samples from a set of HIV-infected subjects soon after influenza vaccination, who we reasoned should have a highly complex immune response. We found that the repertoire patterns of these individuals converged to a common antibody response that is distinct from the repertoires of healthy donors. Our repertoire fingerprinting approach is not completely novel - PCA has been used in previous studies in many different contexts to analyze immune repertoires [21–23]. However, the power of our approach is that we show that the resulting PCA-transformed groups can differentiate repertoires based on disease state, extending the applicability of this technique.

## Results

**Fig. 1:**
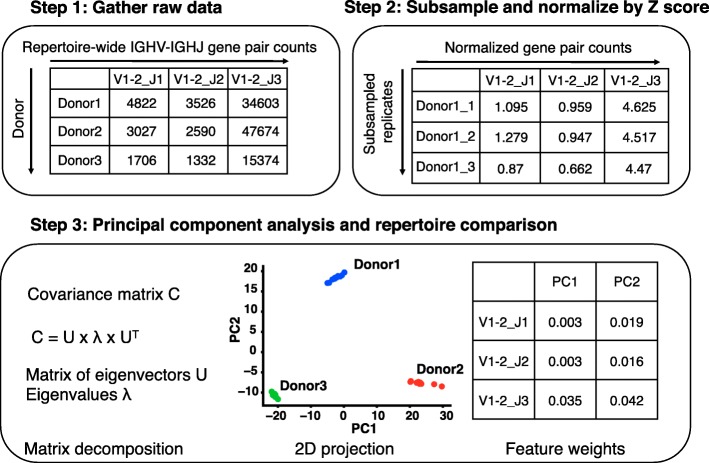
Workflow of repertoire fingerprinting by principal component analysis. To perform repertoire fingerprinting we first sequenced antibody genes of human donors and tabulated the IGHV-IGHJ gene pair usages. We then processed the data by subsampling to uniform depth over 10 replicates per donor and normalized counts by Z score transformation. We used PCA to project the input features onto 2 dimensions and analyze gene pairs that contribute to differences between repertoires

We briefly describe our workflow which is depicted in the flowchart in Fig. 1. We first sequenced antibody variable genes from several donors with different disease states and ages (described in detail below). From the raw sequence data, we determined unique V3J clonotypes [4, 5], where clonotypes were defined as sequences encoded by the same heavy chain Variable (V) and Joining (J) germline genes (henceforth referred to as IGHV and IGHJ respectively) with identical CDRH3 amino acid sequences. Using the distribution of unique V3J clonotypes from each donor, we tabulated IGHV and IGHJ gene usage (henceforth referred to as V-J gene pairs). This resulted in a total of 306 unique V-J gene pairs which comprised our feature data. We then generated replicates of each sequencing dataset by repeated subsampling of V-J gene pairs from the empirical distribution, to a depth of 10^5^ gene pairs per replicate, creating 10 replicates for each data set from each donor. This approach was used to overcome differences in sampling depth between datasets and is similar to the approach in Bolen et al. [17]. In addition, the subsampling was designed to simulate the error introduced when repeatedly sequencing a subject, specifically in rarely observed germline genes. The resulting data matrix containing V-J gene pair counts from subsampled replicates was then normalized according to their Z score (see Methods for details). PCA was then performed on the subsampled replicate data across multiple donors.

**Fig. 2:**
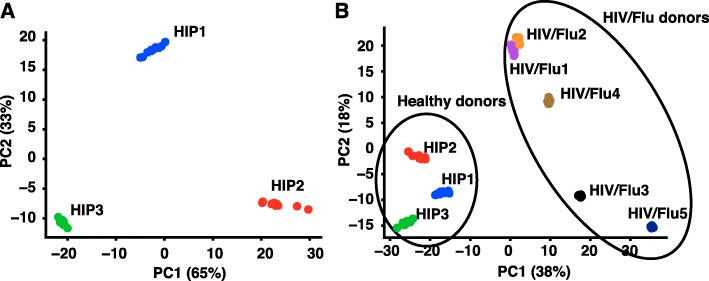
Principal component analysis can be used to distinguish antibody repertoires. **a**. PCA was applied to features of V-J gene use frequency for three healthy donors (HIP1–3). Artificial replicates were generated by subsampling each repertoire to a common depth, repeated 10 times per repertoire. X and Y axes show principal components 1 and 2, and the percent variation explained by each component is shown in parenthesis. **b**. PCA was applied to gene use frequencies from three healthy donors (HIP1–3) and five HIV-infected donors after influenza vaccination (HIV/Flu1–5). Black circles show results of K-means clustering with k = 2 clusters

As a proof of concept, we first applied this methodology to the repertoires of three healthy donors (designated HIP1–3) whose samples were sequenced to extraordinary depth [5]. We found that each donor had a distinct V-J gene pair pattern that could be represented with as few as two principal components while maintaining > 95% of variation in the data (Fig. 2a). We also observed that these donors could not be distinguished by CDRH3 amino acid sequence length, another commonly used feature in antibody repertoires (Additional file 1: Figure S1). From this experiment, we concluded that the input feature space of 306 V-J gene pairs can be reduced significantly to distinguish antibody repertoires from different donors.

Next, we hypothesized that PCA transformation could differentiate repertoires based on disease state. We compared the three healthy subject repertoires HIP1–3 to repertoires from samples obtained from five HIV-positive individuals on day 7 after influenza vaccination (designated “HIV/Flu”; Table 1).

**Table 1:** HIV-infected subjects studied on day 7 after influenza vaccination

HIV/Flu Subject	Race	Ethnicity	Age range (years)	Site of collection
1	Caucasian	Non-Hispanic	50–59	Nashville, TN
2	Caucasian	Non-Hispanic	50–59	
3	Caucasian	Hispanic	30–39	
4	African-American	Non-Hispanic	50–59	
5	Caucasian	Non-Hispanic	40–49	

It was expected that these patients would have an abundance of B cells producing antibodies targeting HIV resulting from chronic infection, as well as a large proportion of circulating plasmablasts stimulated by seasonal influenza vaccination. We isolated PBMCs from the five donors and sequenced their antibody repertoires to analyze the repertoire fingerprints. We found that the first three components could account for 78% of total variation, with 56% in the first two components (Fig. 2b). In addition, we observed that the HIV/Flu repertoires segregated from the healthy repertoires in 2D PC space. We performed K-means clustering on the repertoires transformed into PC1 + 2 space and found that the repertoires clustered based on disease state (Fig. 2b; black circles). Since we had a priori knowledge of two distinct disease states, we reasoned that K-means clustering with just two clusters was a logical choice.

To determine which V-J gene pairs contributed most significantly to the observed differences in HIV/Flu vs. healthy populations, we analyzed the feature weights from principal components 1 and 2 trained on healthy and HIV/Flu donors and plotted them as a heat map (Additional file 1: Figure S2, panel A). We observed that the genes that most strongly contributed to differences in these sets of repertoires were among the most highly expressed antibody heavy chain gene segments in humans [24]. This was not a surprising outcome, considering our normalization method was designed to de-emphasize the contribution of genes with very low counts. Many gene pairs contributed to component 1, including a strong contribution from gene *IGHJ4*. In addition, many IGHV3 family genes appeared to contribute to the HIV/Flu-specific repertoire. Component 2 had strong contributions from *IGHV3–30-3* and *IGHJ4*. This analysis suggests that usage of genes in the IGHV3 family and gene *IGHJ4* was perturbed in the HIV/Flu repertoires. This finding agreed with previous reports that show that *IGHJ4* usage is highly enriched in many memory B cell subsets [7, 25].

To examine whether raw germline gene usage can provide the same level of differentiation, we plotted germline gene usage of two of the V-J gene pairs mostly highly implicated in the PCA, *IGHV3–30-3*_*IGHJ4* and *IGHV4-31*_*IGHJ4* (Additional file 1: Figure S2, panel B). Although there is some differentiation between healthy and HIV/Flu repertoires, it is not nearly as robust as that seen when using PCA. Therefore, we conclude that a PCA of the full germline gene usage data is necessary for robust discrimination between disease states, and that analysis of the top germline genes is not sufficient.

As a control, we investigated the use of alternate features to describe these immune repertoires, including commonly used features such as CDRH3 length, CDRH3 net charge, and CDRH3 amino acid composition. We calculated each of these three features for healthy and HIV/Flu donors and reduced them to two components using the same PCA procedure as previously described. Surprisingly, these variables did not seem to provide added value in distinguishing healthy donors from HIV/Flu donors (Additional file 1: Figure S3). There was no clear separation of donors in principal component space, and the raw values of these features did not appear to differ between healthy and infected/immunized donors. Therefore, we concluded that V-J gene pairing data provides the most information when attempting to distinguish immune repertoires.

**Fig. 3:**
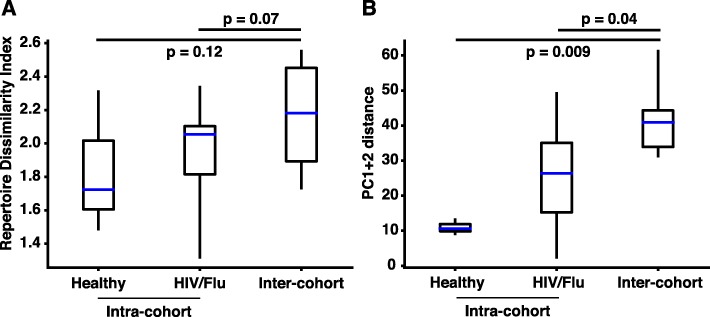
PCA provides better discrimination between donor cohorts than an alternate method. **a.** The Repertoire Dissimilarity Index from Bolen, et al. [17] was calculated for all pairs of donors within both the healthy and HIV/Flu cohorts and for inter-cohort pairs. **b**. The Euclidean distance between principal components (PC) 1 + 2 was calculated for the same intra- and inter-cohort pairs. Boxes show the interquartile range of data, with the median shown in blue, and whiskers show the full range of data. Significance was calculated using a two-sided Mann-Whitney rank test

To test the advantage of our repertoire fingerprinting method compared to an existing approach, we implemented the Repertoire Dissimilarity Index (RDI) metric from Bolen et al. [17]. We then calculated the RDI for each pair of subjects between the healthy cohort and the HIV/Flu cohort and plotted the intra-cohort distance for two subjects in the same cohort (i.e., healthy donor 1 – healthy donor 2), and the inter-cohort distance for subjects in separate cohorts (i.e., healthy donor 1 – HIV/Flu 1) (Fig. 3a). We repeated the same calculation using the Euclidean distance between repertoires in PC space to see which metric provided better discrimination between healthy and infected/immunized donors (Fig. 3b). We observed that, while the RDI provided some separation between intra- and inter-cohort pairs, repertoire fingerprinting provided better separation between these groups (compare Fig. 3a and Fig. 3b). The difference in intra- and inter-cohort groups was not significant (α = 0.05) when comparing either healthy subjects or HIV/Flu donors to inter-cohort pairs using the RDI (*p* = 0.12 and 0.07, respectively). However, when using repertoire fingerprinting the separation between these groups was statistically significant (*p* = 0.009 and 0.04, respectively). Therefore, we conclude that repertoire fingerprinting by PCA provides better discrimination between donor cohorts than using RDI.

**Fig. 4:**
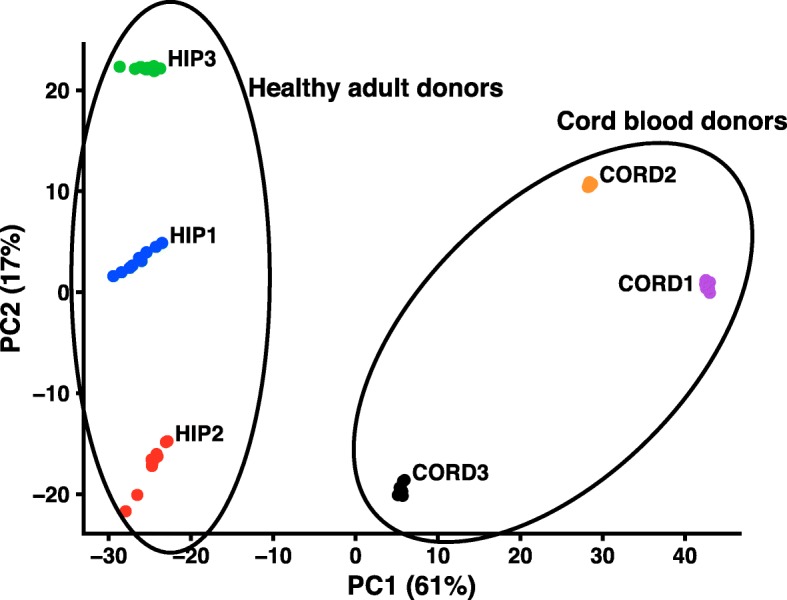
PCA reveals differences between healthy adult and cord blood repertoires. PCA was applied to V-J gene frequency for three healthy donors (HIP1–3) and three cord blood donors (CORD1–3). X and Y axes show principal components 1 and 2, and the percent variation explained by each component is shown in parenthesis. Black circles show results of K-means clustering with k = 2 clusters. Each dot represents a synthetic replicate generated by subsampling each repertoire to a common depth. This subsampling was repeated 10 times per repertoire

We next applied our method to a different comparison of subject groups that differed by age rather than by a recent exposure or infection. We compared immune repertoire fingerprints from cord blood samples of term healthy newborn infants (designated CORD1–3) and compared them with the repertoire of healthy adults (HIP1–3) [5]. We found that these two classes of repertoires that differed by age of subject also could be reliably separated using PCA (Fig. 4). The CORD repertoires showed unique patterns of V-J usage compared to those of healthy adults, with 80% of variation being accounted for in two components. We performed K-means clustering with 2 clusters on these six repertoires in PC1 + 2 space and observed that they separated into healthy adult and cord blood clusters (Fig. 4; black ellipses). To analyze which V-J gene pairs contributed to the differences between adult and cord blood repertoires, we extracted and analyzed the feature weights from PC1 + 2 (Additional file 1: Figure S4). We observed a strong dependence on *IGHJ3* in component 1, and *IGHV3–23* and *IGHV1–69* in component 2 (Additional file 1: Figure S4). In addition, we noticed an upweighting of *IGHV1–2* in component 1, which agreed with previous reports indicating that this gene is highly expressed in cord blood repertoires [7]. The partitioning between the healthy adult and cord blood donor datasets in principal component space provides a clear indication of the utility of this method in distinguishing repertoires based on subjects differing by age.

**Fig. 5:**
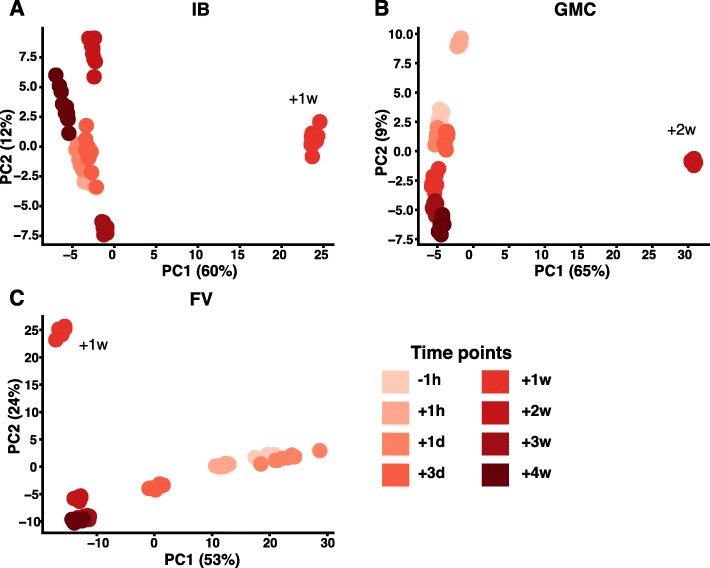
Repertoire fingerprinting by PCA can identify perturbations in repertoire after influenza vaccination. PCA was applied to the sequenced repertoires of three individuals (FV, GMC, IB) at 10 time points before and after seasonal influenza vaccination. Dataset is from Laserson, et al. [26]. Shown are three principal components and the percent variation explained by each in parenthesis. Each dot represents a synthetic replicate generated by subsampling each repertoire to a common depth. This subsampling was repeated 10 times per repertoire

While our method has utility in distinguishing healthy donor data sets, we next wanted to test the method on datasets comprised of immune repertoires in the same individuals before and after an environmental exposure, seasonal influenza vaccination. We used our method on the public datasets from Laserson et al. where the antibody repertoires from three donors (referred to with designations IB, GMC, and FV—author initials, see [26]) were sequenced at ten different time points before and after vaccination. We analyzed data from eight time points, one before vaccination and seven time points after using our PCA-based approach to monitor perturbations in the repertoire corresponding to vaccination. When all time points from all three donors were analyzed in a single PCA, the samples clustered by donor, rather than by time point (i.e. all time points from IB clustered together, those from GMC clustered together, etc.) (data not shown). This finding suggests that the difference in the repertoires between two individuals is greater than the difference in one individual over time, which is an expected finding. Therefore, we performed PCA on each donor separately, to see how the repertoires shifted over time. In each of the three donors, we saw a distinct shift in principal component space shortly after vaccination. In donors IB and GMC there was a distinct shift in V-J usage 1–2 weeks after vaccination, with the repertoire quickly returning to baseline afterwards (Fig. 5a, b). This finding was in agreement with previous studies showing that the influenza-specific portion of the antibody repertoire tends to spike between day 7 and day 30 after vaccination [27, 28]. In the third donor, FV, we observed a more granular view of the dynamics post-vaccination. Perturbations in the repertoire were visible at day 3 post-vaccination, and the repertoire appeared to shift V-J usage significantly for up to 4 weeks post vaccination, which was the last time point recorded (Fig. 5c).

## Discussion

We note several advantages in our method for understanding the complexity of adaptive immune receptor repertoires. Our repertoire fingerprinting method works independent of sequencing depth, as the samples for the healthy donors who formed the main comparator group here were sequenced to extraordinary depth (1.7 × 10^7^ unique clonotypes in sample HIP2, for example) while others were not sequenced as deeply (2.5 × 10^5^ unique clonotypes in sample CORD2, for example). We were able to overcome such large differences in depth by subsampling the repertoires to a common depth. In addition, our method reduces high-dimensional gene use frequency data to just a few components that can be visualized and interpreted easily. By reducing the data into just a few components, the data can be partitioned easily into groups that are most similar. In this way, PCA not only reduces the dimensionality of the analysis problem, but also provides a means for grouping the data in question. In this study, we showed how PCA could be used to distinguish samples from healthy and immunized or disease state donors.

There are at least two possible explanations for the observation that repertoires cluster by disease state. First, it is possible that individuals who share a history of chronic infection (in this study it was HIV) have a convergent response when immunized. A second possibility is that since the cells from the diseased cohort were from day 7 plasmablasts, and the healthy repertoires were derived from both plasmablasts and memory B cells, differences in V-J gene usage might be based purely on differences in the composition of cell phenotypes. From the data we collected, it was not possible to distinguish between these possibilities. Regardless, we concluded that our repertoire fingerprinting method is robust enough to detect differences in V-J gene usage between individuals and represent the difference in reduced feature space.

In this report, we compare our repertoire fingerprinting method to an existing method for comparing immune repertoires, the Repertoire Dissimilarity Index (RDI). Our approach and the RDI are conceptually similar in that they both use subsampling to normalize repertoires by their sequencing depth and use V-J gene usage as inputs. We show that our approach is more robust in distinguishing repertoires by disease state. We believe that our use of PCA to extract the most critical features from the dataset allows us to minimize noise in the datasets and consequently improves differentiation. For this reason, both methods are able to represent the underlying patterns in the dataset, however by removing noisy features our fingerprinting method is able to emphasize the inherent differences in feature space. Ours is not the first study to use PCA to reduce dimensionality of immune repertoire sequencing data [21–23]. However, we believe that this work shows the power of PCA applied to high-dimension sequencing data to distinguish repertoires of different disease states.

Although this repertoire fingerprinting method is promising, we note several caveats. The cohort size of subjects in these groups was small (*n* = 3–5). To overcome the small sample size, we used a subsampling approach to simulate replicates of each sample, which makes our method robust to noise when dealing with a relatively small number of donors. Subsampling provides a spread of data points per donor to assess whether differences are outside the range of error and allows us to conclude that our findings are not an effect of noise. In addition, the sequencing depth varied between disease states (Additional file 2: Table S1). While our subsampling approach was designed to simulate equal sequencing depth between samples, further work is needed to rule out the possibility that sequencing depth affects the PCA-based differentiation we report here. We also acknowledge that our samples are from donors with extreme immunological perturbations (i.e. HIV-positive post-influenza vaccination and newborn cord blood). The signal we detect here may be due to the fact that the donors are in vastly different immunological states. Future research is needed to validate the method on donors with less extreme differences in their repertoire composition. Finally, it was reasonable to assume that HIV- and influenza-specific antibodies were enriched in the HIV/Flu donor samples based on the temporal aspects of sample collection. However, we did not verify the binding specificity of the over-represented antibody clones induced by vaccination or infection. In future studies, we plan to identify repertoire fingerprints specific to an infection and test the binding activity of the enriched clones to confirm their targets.

All data in this study were collected from circulating B cells in peripheral blood. It has been shown that B cells from different tissue compartments have unique patterns of somatic hypermutation and germline gene usage [29]. In this study, we only examined the blood compartment due to the fact that we wanted to focus on repertoire profiling in a way that would be tractable to extend to human donors in the future. However, we anticipate that our repertoire fingerprinting method would be robust to repertoire sequencing data gathered from any tissue.

All data in this study were processed using the same library preparation and sequencing methods to allow a fair comparison. However, we observed that when applying this method to repertoires sequenced using different protocols, the comparison may be confounded by variables such as preferential amplification of one germline family, or there may be an apparent bias in V-J frequencies owing to the sequencing protocol (data not shown). Thus, although this method can be applied to repertoires obtained using any protocol, the results are likely to be most meaningful when comparing repertoires for two samples obtained using the same amplification and sequencing protocols.

In this work we focused on B cell heavy chains, due to the fact that these chains generally dominate the interactions responsible for specific antigen recognition. However, there is no reason why the current methodology couldn’t be applied to T cell receptor beta (TCRβ) chains or to light chains from either immunoglobulin or TCRs. With continued development of paired sequencing methods [30], we believe that the addition of B cell light chain and TCRβ sequences would only increase the signal and allow for better separation of donors into their respective cohorts after PCA transformation. In future work, we plan to add additional genetic features into the repertoire fingerprint.

## Conclusion

In this work, we report a new method called “repertoire fingerprinting” that uses PCA to analyze the frequency of V-J gene pairing and extract two descriptors from a repertoire that can be compared easily across individuals. We show that PCA is sufficient to differentiate healthy donors from one another, independent of sampling depth, and use this analysis to distinguish healthy donors from HIV-positive donors after influenza vaccination. The repertoire fingerprints give a robust discrimination of the health state and shed light on the V and J genes that contribute most to the HIV/influenza response. We extended this analysis to cord blood samples and showed that the methods also exhibit the ability to discriminate repertoires that differ based on subject age. We also validated this method on external sequences from a publicly available dataset studying antibody repertoires after influenza vaccination and found that we can detect dynamic changes in the peripheral blood antibody repertoire after vaccination.

## Methods

### Sample preparation and sequencing

Peripheral blood was obtained from healthy adult donors following written informed consent, under a protocol approved by the Vanderbilt Institutional Review Board. B cells from approximately 1 × 10^7^ PBMCs per donor sample were enriched using EasySep Human Pan-B Cell Enrichment Kit on the RoboSepTM-S according to the manufacturer’s protocol (Stemcell Technologies). After the enrichment, cells were washed and pelleted for total RNA extraction using the RNeasy Mini Kit (Qiagen). First-strand cDNA synthesis was performed by using PrimeScript Reverse Transcriptase (Clontech), following the manufacturer’s instructions (with optional steps), using 20 pmol of J gene-specific primers [31] with unique molecular identifiers incorporated into the 5' end of the primers. After cDNA synthesis, samples were purified using the AmpureXP Size Select Bead Kit (Beckman Coulter). Immediately following bead clean up, 30 μL of PCR mixture containing 2.5 pmol of each V gene-specific region primer [31] and 2X Kapa Hifi Hotstart Ready Mix (Kapa Biosystems) was added directly to the 20 μL purified first-strand synthesis product. PCR reaction conditions were 95 °C for 3 min, 9 cycles of 98 °C for 20 s, 65 °C for 15 s, and 72 °C for 30 s, and a final extension step of 72 °C for 5 min. The first-round PCR reaction was purified using the Ampure Size Select Bead Kit (Beckman Coulter). Second-round PCR mixture containing 25 pmols of each Illumina adapter extension primer and 2X Kapa Hifi Hotstart Ready Mix (Kapa Biosystems) was added directly to 20 μL of the purified first-round PCR reaction product. PCR reaction conditions were 95 °C for 3 min, 23 cycles of 98 °C for 20 s, 65 °C for 15 s, and 72 °C for 20 s, and a final extension step of 72 °C for 5 min. The second-round PCR products were purified using the Ampure Size Select Bead Kit (Beckman Coulter). Illumina-ready amplicon libraries were quantified using the Real-time Library Amplification Kit (Kapa Biosystems) and pooled at equimolar amounts. Samples were loaded onto 2X flow cells for sequencing on the HiSeq 2500 next-generation sequencer with PE-250 V2 chemistry (Illumina).

### Data processing and analysis

All V3J clonotypes from the HIP and CORD data sets were obtained directly from [5]. A similar approach as described in [5] was used to process the HIV/Flu samples and is briefly described below. The processing pipeline consisted of the following steps. First, the FASTQC [32] toolkit was used to inspect the quality of the run. Next, full-length reads were generated from Illumina paired-end reads using the software package USEARCH (version 9.1) [33]; 3) The BIOMEDII primers (Additional file 2: Table S2) were removed using the software package FLEXBAR (version 3.0) [34]. Data were then processed using the PyIR informatics pipeline (https://github.com/crowelab/PyIR)) and the resulting sequences filtered based on the following criteria: 1) the E value had to be less than 10^− 6^ for both the V and J germline alignments; 2) the junctional sequence was in-frame; 3) the junctional sequence was productive; 4) the sequence did not contain stop codons; and 5) a CDR3 sequence was defined. We did not filter any of the sequences based on Phred scores (as in [5]). Unique V3J clonotypes were obtained from all the remaining sequences belonging to a specific donor. The frequency of each V-J gene pair was computed from the V3J clonotypes belonging to each individual donor. Data from Laserson et al. [26] was processed in a similar manner.

### Normalization and principal component analysis

Datasets first were subsampled with replacement to 10^5^ sequences to account for differences in sequencing depth, similar to the method reported in Bolen, et al. [17]. Each dataset was subsampled 10 times to account for noise in the datasets and to reduce the possibility of overfitting to a small number of donors. We reduced the full sequence data set to a subset of 306 common V-J pairs before performing subsampling (Additional file 2: Table S3).

V-J counts were normalized using a Z score normalization method, as in [35]. Briefly, the counts were first log_10_ transformed to account for large differences in gene counts, using a pseudocount of 0.01 for genes that were never observed. The values were converted to a Z score by subtracting the mean and dividing by the standard deviation. The Z scores then were transformed out of log space before principal component analysis. We observed that this step improved performance by de-emphasizing the contribution of genes with very low or no counts in the sequences.

To investigate the use of alternate features to describe repertoires, we calculated CDRH3 length, overall CDRH3 charge, and CDRH3 amino acid usage in healthy and HIV/Flu repertoires. We grouped CDRH3 length and charge into discrete bins, from length 4 to 30 and charge − 6 to + 6. These three characteristics were used as input to a PCA model, from which the top two principal components were extracted and used for comparison. We chose to use mean amino acid frequency rather than positional amino acid frequency due to the difficulty in building a position-specific matrix for variable CDRH3 lengths. During subsampling of the V-J gene pair data, we observed that the amount of variation was roughly equal to 5% per V-J bin. Therefore, rather than subsampling to generate replicates, as in the V-J gene pair data, we directly added 5% gaussian noise to each of the length, charge, and amino acid composition bins to simulate replicates.

Principle component analysis (PCA) is a dimensionality reduction technique that transforms input data into orthogonal components that maximize the variance in the transformed data. PCA was performed using the scikit-learn package in Python [36]. PCA-transformed data were plotted using the Matplotlib library [37]. To infer clusters from PCA-transformed data, we used K-means clustering to determine which data points constituted a cluster. K-means clustering was performed using the scikit-learn package [36] with two clusters (K = 2).

## Supplementary information

**Additional file 1: Figure S1.** Histogram of CDRH3 lengths for three healthy donors (HIP1–3). **Figure S2.** A. PCA was applied to healthy and HIV/Flu donors and the feature weights of each V-J gene pair are shown as a heat map for principal component 1 (left) and 2 (right). B. Germline gene usage of two V-J gene pairs are plotted for each of the 8 datasets. X- and Y-axes show normalized gene usage. **Figure S3.** Use of alternate features to describe the HIV/Flu repertoire do not distinguish healthy from infected donors. Building a PCA model based on CDRH3 length, charge, and amino acid composition (A) does not provide separation between healthy and infected repertoires. Raw distributions of these features are shown in panels B, C, and D. **Figure S4.** PCA was applied to healthy adults and cord blood donors and the feature weights of each V-J pair are shown as a heat map for principal component 1 (left) and 2 (right).

**Additional file 2: Table S1**. Number of unique clonotypes analyzed for each of the 11 donors. **Table S2**. 306 common V-J pairs were used to perform normalization and PCA transformation, to reduce the contribution from rare genes. These genes are listed below. **Table S3**: BIOMEDII primers

## Data Availability

The dataset(s) supporting the conclusions of this article is (are) available in the Sequence Read Archive (SRA) under Bioproject number PRJNA511481 https://www.ncbi.nlm.nih.gov/bioproject/PRJNA511481/ (for HIP data) and PRJNA553768 (for HIV/influenza data). Software used in computing the immune repertoire fingerprints can be downloaded from the following Github repository: http://github.com/crowelab/Fingerprint

## Abbreviations

CDR3: Heavy chain complementarity determining region
HIV/Flu: Cohort of HIV-positive donors after seasonal influenza vaccination
NGS: Next-generation sequencing
PCA: Principle component analysis
V-J: Variable (V) and joining (J) immunoglobulin germline gene assignment
