# Supplementary material for: Immune repertoire fingerprinting by principal component analysis reveals shared features in subject groups with common exposures
Source: BMC Bioinformatics. 2019 Dec 4;20:629. doi: 10.1186/s12859-019-3281-8 (PMC6894320; doi:10.1186/s12859-019-3281-8)

Figure S1. Histogram of CDRH3 lengths for three healthy donors (HIP1-3).

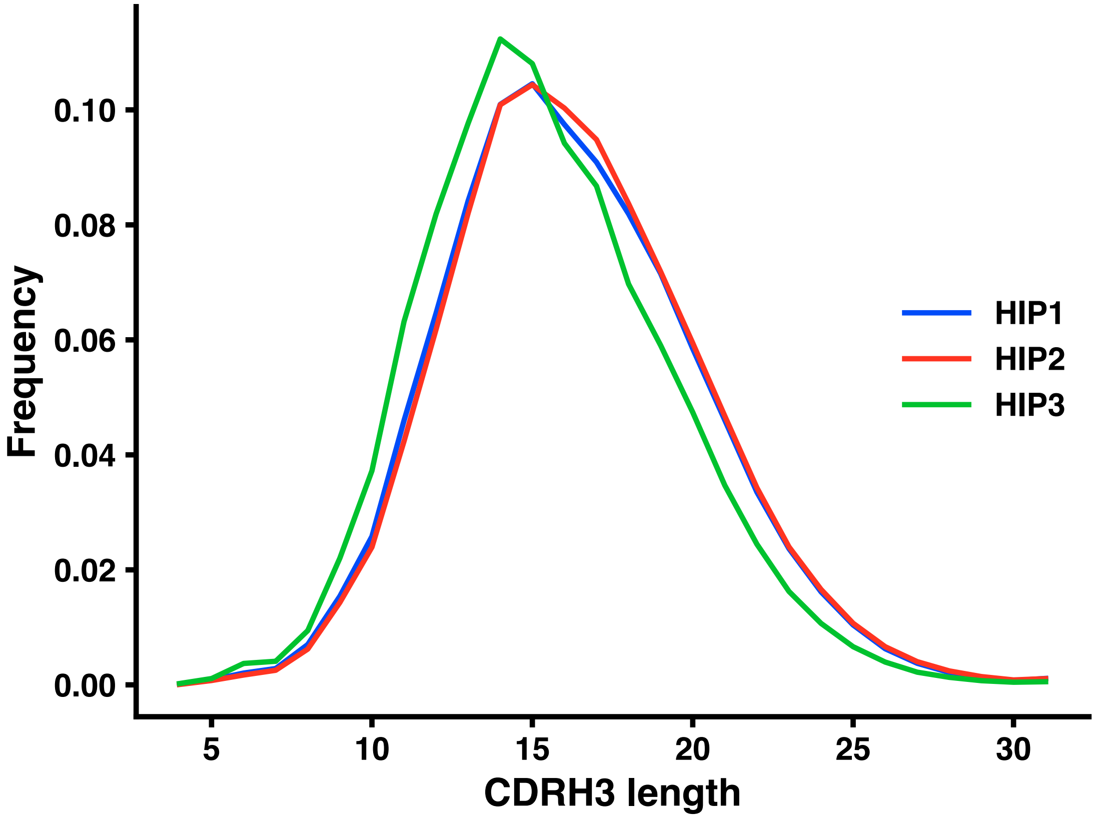

Figure S2. A. PCA was applied to healthy and HIV/Flu donors and the feature weights of each V-J gene pair are shown as a heat map for principal component 1 (left) and 2 (right). B. Germline gene usage of two V-J gene pairs are plotted for each of the 8 datasets. X- and Y-axes show normalized gene usage.

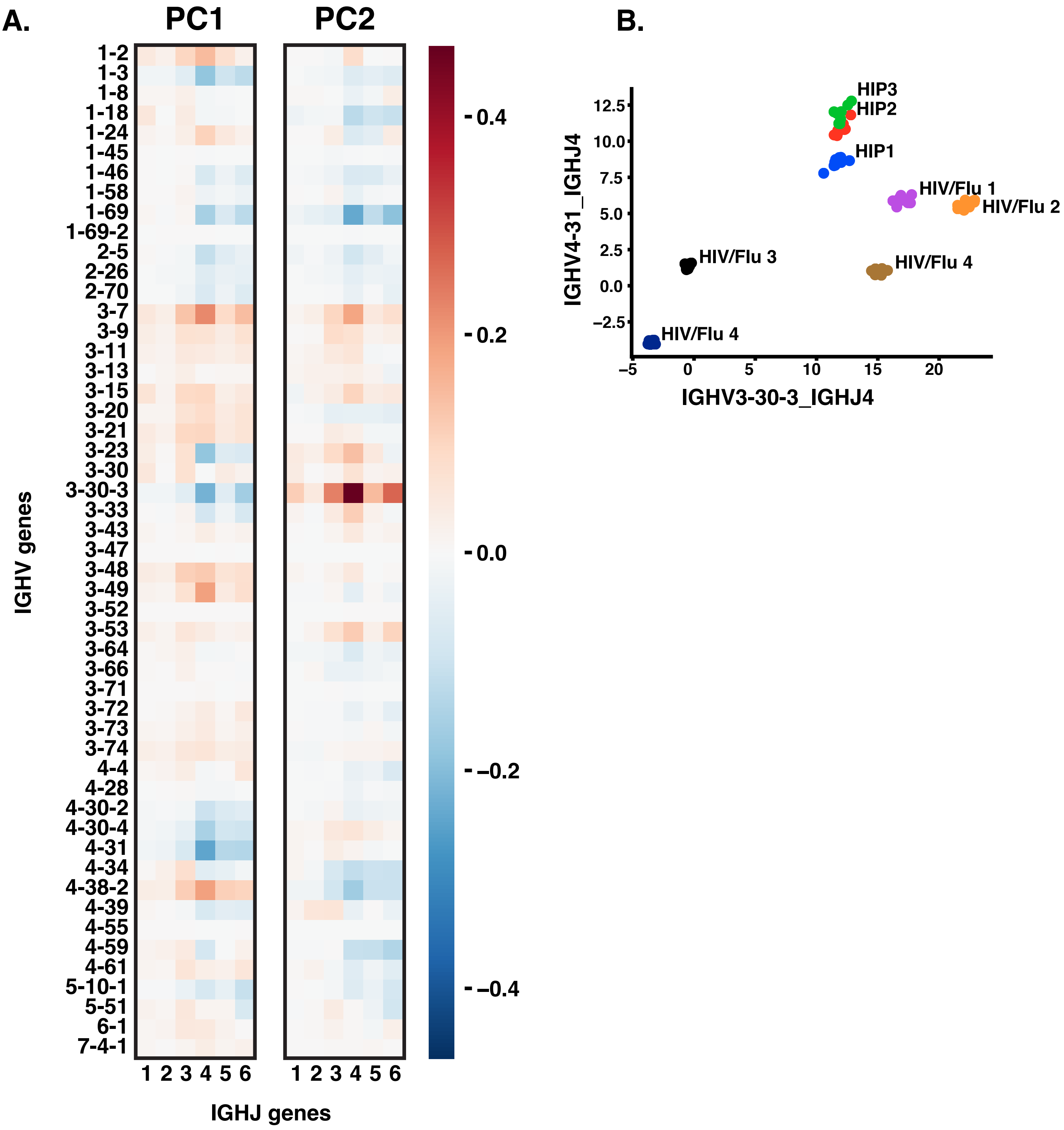

Figure S3. Use of alternate features to describe the HIV/Flu repertoire do not distinguish healthy from infected donors. Building a PCA model based on CDRH3 length, charge, and amino acid composition (A) does not provide separation between healthy and infected repertoires. Raw distributions of these features are shown in panels B, C, and D.

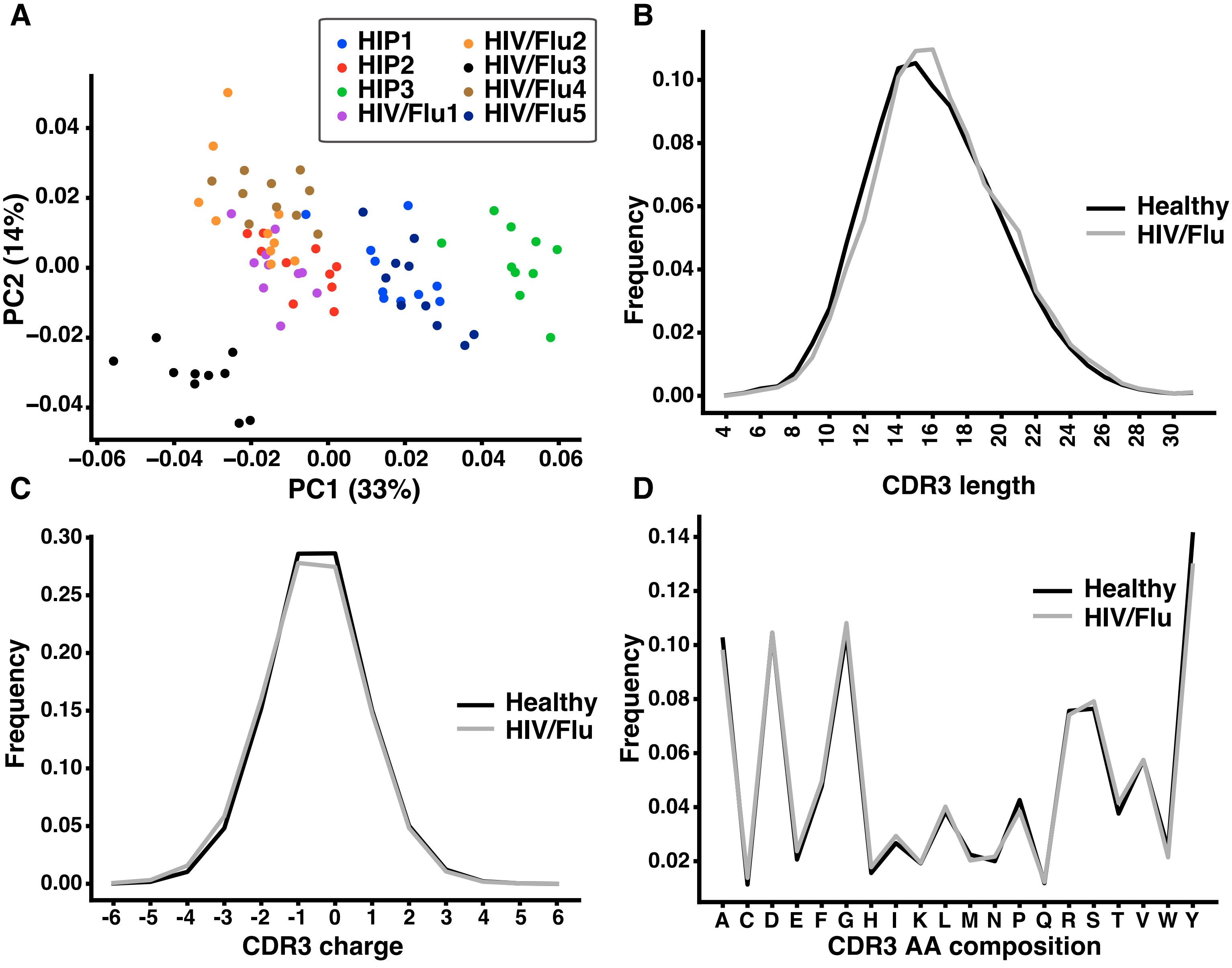

Figure S4. PCA was applied to healthy adults and cord blood donors and the feature weights of each V-J pair are shown as a heat map for principal component 1 (left) and 2 (right).

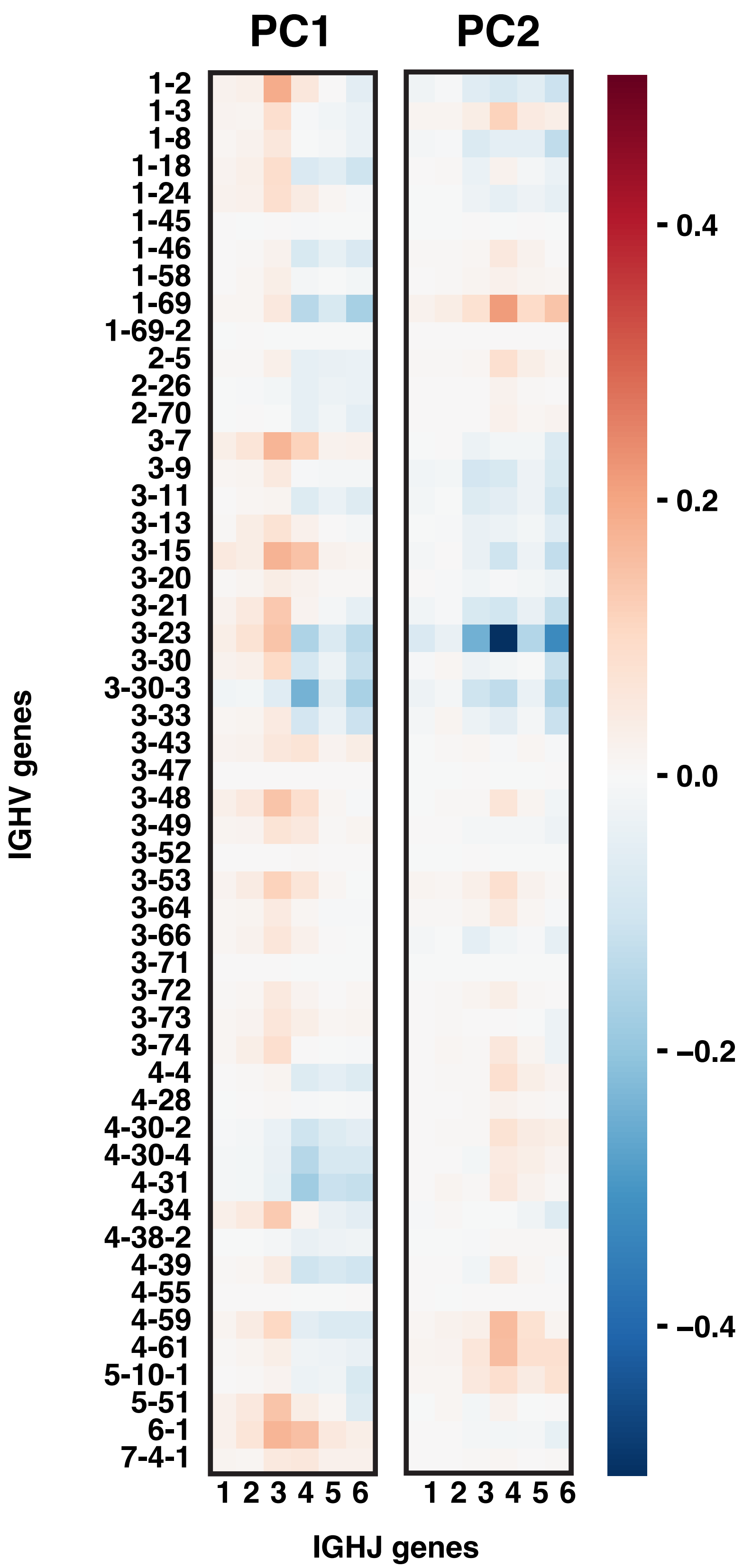

Supplement: Supplementary file 1 — Additional file 1: Figure S1. Histogram of CDRH3 lengths for three healthy donors (HIP1–3). Figure S2. A. PCA was applied to healthy and HIV/Flu donors and the feature weights of each V-J gene pair are shown as a heat map for principal component 1 (left) and 2 (right). B. Germline gene usage of two V-J gene pairs are plotted for each of the 8 datasets. X- and Y-axes show normalized gene usage. Figure S3. Use of alternate features to describe the HIV/Flu repertoire do not distinguish healthy from infected donors. Building a PCA model based on CDRH3 length, charge, and amino acid composition (A) does not provide separation between healthy and infected repertoires. Raw distributions of these features are shown in panels B, C, and D. Figure S4. PCA was applied to healthy adults and cord blood donors and the feature weights of each V-J pair are shown as a heat map for principal component 1 (left) and 2 (right). [file 12859_2019_3281_MOESM1_ESM.pdf]
