# Supplementary material for: Immune repertoire fingerprinting by principal component analysis reveals shared features in subject groups with common exposures
Source: BMC Bioinformatics. 2019 Dec 4;20:629. doi: 10.1186/s12859-019-3281-8 (PMC6894320; doi:10.1186/s12859-019-3281-8)
Supplement: Supplementary file 2 — Additional file 2: Table S1. Number of unique clonotypes analyzed for each of the 11 donors. Table S2. 306 common V-J pairs were used to perform normalization and PCA transformation, to reduce the contribution from rare genes. These genes are listed below. Table S3: BIOMEDII primers [file 12859_2019_3281_MOESM2_ESM.pdf]

Table S1. Number of unique clonotypes analyzed for each of the 11 donors.

Donor	# unique clonotypes
HIP1	10,706,238
HIP2	17,102,271
HIP3	8,982,311
HIV/Flu1	402,288
HIV/Flu2	414,420
HIV/Flu3	645,147
HIV/Flu4	447,724
HIV/Flu5	314,140
CORD1	231,205
CORD2	248,023
CORD3	323,762

Table S2. 306 common V-J pairs were used to perform normalization and PCA transformation, to reduce the contribution from rare genes. These genes are listed below.

**V genes**	**V genes cont'd**	**J genes**
IGHV1-2	IGHV3-48	IGHJ1
IGHV1-3	IGHV3-49	IGHJ2
IGHV1-8	IGHV3-52	IGHJ3
IGHV1-18	IGHV3-53	IGHJ4
IGHV1-24	IGHV3-64	IGHJ5
IGHV1-45	IGHV3-66	IGHJ6
IGHV1-46	IGHV3-71	
IGHV1-58	IGHV3-72	
IGHV1-69	IGHV3-73	
IGHV1-69-2	IGHV3-74	
IGHV2-5	IGHV4-4	
IGHV2-26	IGHV4-28	
IGHV2-70	IGHV4-30-2	
IGHV3-7	IGHV4-30-4	
IGHV3-9	IGHV4-31	
IGHV3-11	IGHV4-34	
IGHV3-13	IGHV4-38-2	
IGHV3-15	IGHV4-39	
IGHV3-20	IGHV4-55	
IGHV3-21	IGHV4-59	
IGHV3-23	IGHV4-61	
IGHV3-30	IGHV5-10-1	
IGHV3-30-3	IGHV5-51	
IGHV3-33	IGHV6-1	
IGHV3-43	IGHV7-4-1	
IGHV3-47		

Table S3: BIOMEDII primers

Primer	Application	Sequence
Human IgH cDNA synthesis and reverse PCR primer		
J_H	Human IgH RT primer and reverse PCR primer	NNNNCTTACCTGAGGAGACGGTGACC
Human IgH forward PCR primer mix		
V_H1-FR1	Human multiplex forward IgH PCR primer	NNNNGGCCTCAGTGAAGGTCTCCTGCAAG
V_H2-FR1	Human multiplex forward IgH PCR primer	NNNNGTCTGGTCCTACGCTGGTGAACCC
V_H3-FR1	Human multiplex forward IgH PCR primer	NNNNCTGGGGGGTCCCTGAGACTCTCCTG
V_H4-FR1	Human multiplex forward IgH PCR primer	NNNNCTTCGGAGACCCTGTCCCTCACCTG
V_H5-FR1	Human multiplex forward IgH PCR primer	NNNNCGGGGAGTCTCTGAAGATCTCCTGT
V_H6-FR1	Human multiplex forward IgH PCR primer	NNNNTCGCAGACCCTCTCACTCACCTGTG
Human IgK cDNA synthesis and reverse PCR primer mix		
J_K1	Human IgK RT primer and reverse PCR primer	NNNNTTTGATATCCACCTTGGTCCC
J_K2	Human IgK RT primer and reverse PCR primer	NNNNTTTAATCTCCAGTCGTGTCCC
Human IgK forward PCR primer mix		
V_K1-2-FR1	Human multiplex forward IgK PCR primer	NNNNATGAGGSTCCCYGCTCAGCTGCTGG
V_K3-FR1	Human multiplex forward IgK PCR primer	NNNNCTCTTCCTCCTGCTACTCTGGCTCCCAG
V_K4-FR1	Human multiplex forward IgK PCR primer	NNNNATTTCTCTGTTGCTCTGGATCTCTG
Human Igλ cDNA synthesis and reverse PCR primer mix		
J_λ1	Human Igλ RT primer and reverse PCR primer	NNNNAGGACGGTGACCTTGGTCCC
J_λ2	Human Igλ RT primer and reverse PCR primer	NNNNAGGACGGTCAGCTGGGTCCC
Human Igλ forward PCR primer mix		
V_λ1-FR1	Human multiplex forward Igλ PCR primer	NNNNGGTCCTGGGCCCAGTCTGTGCTG
V_λ2-FR1	Human multiplex forward Igλ PCR primer	NNNNGGTCCTGGGCCCAGTCTGCCCTG
V_λ3-FR1	Human multiplex forward Igλ PCR primer	NNNNGCTCTGTGACCTCCTATGAGCTG
V_λ4+5-FR1	Human multiplex forward Igλ PCR primer	NNNNGGTCTCTCTCSCAGCYTGTGCTG
V_λ6-FR1	Human multiplex forward Igλ PCR primer	NNNNGTTCTTGGGCCAATTTTATGCTG
V_λ7-FR1	Human multiplex forward Igλ PCR primer	NNNNGGTCCAATTCYCAGGCTGTGGTG
V_λ8-FR1	Human multiplex forward Igλ PCR primer	NNNNGAGTGGATTCTCAGACTGTGGTG
